# Antimicrobial Activity of Cyclic-Monomeric and Dimeric Derivatives of the Snail-Derived Peptide Cm-p5 against Viral and Multidrug-Resistant Bacterial Strains

**DOI:** 10.3390/biom11050745

**Published:** 2021-05-17

**Authors:** Melaine González-García, Fidel Morales-Vicente, Erbio Díaz Pico, Hilda Garay, Daniel G. Rivera, Mark Grieshober, Lia Raluca Olari, Rüdiger Groß, Carina Conzelmann, Franziska Krüger, Fabian Zech, Caterina Prelli Bozzo, Janis A. Müller, Alexander Zelikin, Heinz Raber, Dennis Kubiczek, Frank Rosenau, Jan Münch, Steffen Stenger, Barbara Spellerberg, Octavio L. Franco, Armando A. Rodriguez Alfonso, Ludger Ständker, Anselmo J. Otero-Gonzalez

**Affiliations:** 1 Center for Protein Studies, Faculty of Biology, University of Havana, 25 St, La Habana 10400, Cuba; mgonzalez@fbio.uh.cu; 2 General Chemistry Department, Faculty of Chemistry and Center for Natural Products Research, Faculty of Chemistry, University of Havana, Zapata street, La Habana 10400, Cuba; femvicente@gmail.com; 3 Synthetic Peptides Group, Center for Genetic Engineering and Biotechnology, P.O. Box 6162, La Habana 10600, Cuba; erbio.diaz@cigb.edu.cu (E.D.P.); hilda.garay@cigb.edu.cu (H.G.); 4 Faculty of Chemistry, Havana University, Zapata Street, La Habana 10400, Cuba; dgr@fq.uh.cu; 5 Institute of Medical Microbiology and Hygiene, University Clinic of Ulm, Albert-Einstein-Allee 23, 89081 Ulm, Germany; mark.grieshober@uniklinik-ulm.de (M.G.); Steffen.stenger@uniklinik-ulm.de (S.S.); barbara.spellerberg@uni-ulm.de (B.S.); 6 Institute of Molecular Virology, University Clinic of Ulm, Meyerhofstrasse. 1, 89081 Ulm, Germany; lia-raluca.olari@uni-ulm.de (L.R.O.); ruediger.gross@uni-ulm.de (R.G.); carina.conzelmann@uni-ulm.de (C.C.); franziska.krueger@uni-ulm.de (F.K.); fabian.zech@uni-ulm.de (F.Z.); caterina.prelli-bozzo@uni-ulm.de (C.P.B.); janis.mueller@uni-ulm.de (J.A.M.); jan.muench@uni-ulm.de (J.M.); 7 Department of Chemistry, Aarhus University, Langelandsgade 140, 8000 Aarhus, Denmark; zelikin@chem.au.dk; 8 Institute of Pharmaceutical Biotechnology, Ulm University, 89081 Ulm, Germany; Heinz.Raber@uni-ulm.de (H.R.); dennis.kubiczek@uni-ulm.de (D.K.); frank.rosenau@uni-ulm.de (F.R.); 9 Core Facility for Functional Peptidomics, Ulm Peptide Pharmaceuticals (U-PEP), Faculty of Medicine, Ulm University, 89081 Ulm, Germany; armando.rodriguez-alfonso@uni-ulm.de; 10 Department of Biotechnology, Catholic University Dom Bosco, Campo Grande and Center for Biochemical and Proteomics Analyses, Catholic University of Brasilia, Brasilia, DF 71966-700, Brazil

**Keywords:** antimicrobial peptides, Cm-p5, antibacterial activity, multiresistant microorganisms, chemical derivatives

## Abstract

Cm-p5 is a snail-derived antimicrobial peptide, which demonstrated antifungal activity against the pathogenic strains of *Candida albicans*. Previously we synthetized a cyclic monomer as well as a parallel and an antiparallel dimer of Cm-p5 with improved antifungal activity. Considering the alarming increase of microbial resistance to conventional antibiotics, here we evaluated the antimicrobial activity of these derivatives against multiresistant and problematic bacteria and against important viral agents. The three peptides showed a moderate activity against *Pseudomonas aeruginosa*, *Klebsiella pneumoniae* *Extended Spectrum β-Lactamase* (ESBL), and *Streptococcus agalactiae*, with MIC values > 100 µg/mL. They exerted a considerable activity with MIC values between 25–50 µg/mL against *Acinetobacter baumanii* and *Enterococcus faecium*. In addition, the two dimers showed a moderate activity against *Pseudomonas aeruginosa* PA14. The three Cm-p5 derivatives inhibited a virulent extracellular strain of *Mycobacterium tuberculosis*, in a dose-dependent manner. Moreover, they inhibited Herpes Simplex Virus 2 (HSV-2) infection in a concentration-dependent manner, but had no effect on infection by the Zika Virus (ZIKV) or pseudoparticles of Severe Acute Respiratory Syndrome Corona Virus 2 (SARS-CoV-2). At concentrations of >100 µg/mL, the three new Cm-p5 derivatives showed toxicity on different eukaryotic cells tested. Considering a certain cell toxicity but a potential interesting activity against the multiresistant strains of bacteria and HSV-2, our compounds require future structural optimization.

## 1. Introduction

Cm-p5 is an antimicrobial peptide composed of 12 amino acid residues (SRSELIVHQRLF-NH_2_, PDB ID: 2MP9, BMRB access number: 19973) with an α-helical structure in membrane–mimetic conditions and a tendency to a random structure in aqueous solutions. This peptide was synthesized using Cm-p1 as a template, in order to improve its antifungal activity. Cm-p1 is a cationic peptide of 10 amino acid residues (SRSELIVHQR) derived from the marine snail *Cenchritis muricatus*, Linnaeus, 1758 (Gastropoda: Littorinidae). In previous studies, Cm-p5 showed antifungal activity against the pathogenic yeast species *Candida albicans* and *Candida parapsilosis* and did not exhibit hemolytic activity or any toxic effect against mammalian cell lines in vitro [1,2].

**Figure 1:**
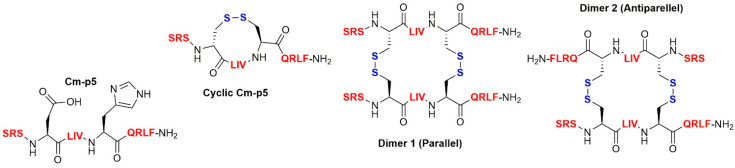
Structure of the designed cyclic analogues of Cm-p5.

Despite its potential clinical benefits, Cm-p5 might not be a suitable therapeutic agent, due to its proteolytic instability and lower activity, as compared to conventional antifungals. We therefore synthesized a cyclic monomer and two dimers (parallel and antiparallel) of Cm-p5 through the introduction of disulfide bonds (Figure 1). These novel derivatives revealed a helical stabilization with improved antifungal activity for the cyclic monomer and an unexpected antibacterial activity against *Pseudomonas aeruginosa* and *Listeria monocytogenes* for the antiparallel dimer. In addition, neither the cyclic monomer nor the dimers were toxic. The antimicrobial activity, the low cytotoxicity, and the stabilization of the α-helix of these derivatives, suggest a greater metabolic stability and activity in vivo [3].

The emergence of multiresistant pathogens is one of the greatest threats to humanity. It represents a tremendous challenge for public health in the near future, and there is an urgent need for alternative antimicrobial substances [4]. In this context, antimicrobial peptides (AMPs), represent promising candidates for the treatment of infectious diseases [5]. The therapeutic potential of AMPs is mainly because they have a broad spectrum of activity against various microorganisms (fungi, bacteria, viruses, and parasites), exert potential in vitro activity against multiresistant strains, and are generally slightly toxic to host cells [6]. Moreover, development of resistance against AMP is unlikely to occur [7]. Additionally, they have a wide distribution in nature and serve an important function as effectors of innate immunity in invertebrates [8], so not only can they act directly on microorganisms but also indirectly through the innate immune system activation [9].

There are many sources of AMPs according to the Antimicrobial Peptide Database (https://wangapd3.com/main.php) but chemical or biotechnological derivatives are reported as a practical and effective source of modified peptides with increased antimicrobial activity or decreased in vitro cytotoxicity [10].

In this work, we enlarge the bacterial and viral spectrum in order to explore the antimicrobial activity of Cm-p5 and its derivatives against viral and multiresistant or problematic bacterial strains.

## 2. Materials and Methods

### 2.1. Bacterial Strains and Growth Conditions

We used the following bacterial species: *Streptococcus agalactiae* ATCC 12403, *Staphylococcus aureus* MRSA ATCC 43300, *Klebsiella pneumoniae* Extended Spectrum β-Lactamase (ESBL) ATCC 700603, *Acinetobacter baumanii* ATCC19606, and *Enterococcus faecium* (VRE) DSM17050. Bacteria were cultured at 37 °C/5% CO_2_ in liquid THY broth (Todd-Hewitt Broth, Oxoid, Hampshire, UK) supplemented with 0.5% yeast extract (BD, Miami, FA, USA). *Pseudomonas aeruginosa PA14* (DSM 19882) was cultivated in liquid Müller–Hinton–Broth (Carl Roth, Karlsruhe, Germany) at 37 °C, under orbital shaking at 160 rpm.

In addition, the employed *Mycobacterium tuberculosis* ATCC 27294 (Mtb) was grown in 7H9-medium containing 7H9 BBL Middlebrook broth (BD), glycerol (Honeywell, Charlotte, NC, USA) OADC (Oelic Albumin Dextrose Catalase; BD), Tween 80 (Roth, Karlsruhe, Germany), and ddH_2_O. The pH was adjusted between 7.2–7.4 and sterile filtration was performed with a 0.2-µm filter membrane (Thermo Scientific^TM^ Nalgene^TM^ Rapid-Flow^TM^, Thermo Scientific, Bremen, Germany).

### 2.2. Mammalian Cell Lines and Culture Conditions

Vero E6 (*Cercopithecus aethiops* derived epithelial kidney) cells were grown in Dulbecc’s modified Eagle’s medium (DMEM) supplemented with 2.5% heat-inactivated fetal calf serum (FCS), 2 mM L-glutamine, 100 units/mL penicillin, 100 µg/mL streptomycin, 1 mM sodium pyruvate, and non-essential amino acids (Sigma #M7145, St. Louis, MI, USA). HEK293T cells are a human cell line originally derived from embryonic kidney. Transformed by adenovirus type 5 expressing SV40 (simian virus 40) large T-antigen [11]. ELVIS™ (Enzyme-Linked Virus-Inducible System) are a baby hamster kidney cell line encoding lacZ gene, which is expressed upon infection via viral transactivator ICP10 [12].

HEK293T and ELVIS™ cells were cultured in DMEM supplemented with 2 mM L-glutamine, 100 units/mL penicillin, and 100 μg/mL streptomycin, and 10% heat-inactivated FCS. Caco-2 (human epithelial colorectal adenocarcinoma) cells were grown in DMEM supplemented with 20% FCS, 100 units /mL penicillin, 100 µg/mL streptomycin, 1 mM sodium pyruvate, and non-essential amino acids (Sigma Aldrich, St. Louis, MO, USA) and seeded in the same medium with only 10% FCS for experiments. Cells were grown at 37 °C in a 5% CO_2_ humidified incubator.

### 2.3. Peptide Synthesis

Solid phase peptide synthesis was based on the use of Fmoc/t-Bu chemistry on rink amide resin, based on polystyrene or PEG (Sigma Aldrich). The resin was washed with DMF (2 × 2 mL, 1 min), DCM (2 × 2 mL, 1 min), MeOH (2 × 2 mL, 1 min), DCM (2 × 2 mL, 1 min), and DMF (2 × 2 mL, 1 min). Fmoc removal was conducted with 20% piperidine in DMF (2 × 10 min), and the subsequent amino acids were added using the coupling condition—Fmoc-Aa-OH/DIC/Oxyma (4 equivalent of each) in DMF, after a negative ninhydrin test (approximately 30 min). All peptides were prepared with more than 95% purity as evaluated by analytical RP-HPLC. The molecular mass was experimentally established by ESI–MS, in correspondence with the theoretically calculated monoisotopic mass for each peptide vivo [3].

### 2.4. Antibacterial Activity

#### 2.4.1. Agar Overlay Assay

Bacteria were cultured at 37 °C/5% CO_2_ overnight, pelleted by centrifugation and washed in 10 mM sodium phosphate buffer. Following resuspension in 10 mM sodium phosphate buffer, optical density was determined at 600 nm. 2 × 10^7^ bacteria were seeded into a petri dish in 1% agarose and 10 mM sodium phosphate buffer. After cooling at 4 °C for 30 min, 3–5 mm holes were cut into the 1% agarose. Peptides adjusted to the desired concentration in 10 µL of buffer were filled into the agar-holes. Following an incubation at 37 °C in ambient air for 3 h, the plates were overlaid with 1% agarose and 3% tryptic soy solved in 10 mM phosphate buffer. Inhibition zones in millimeter were determined following a 16–18 h incubation time at 37 °C/5% CO_2_.

#### 2.4.2. Minimal Inhibitory Concentration (MIC) Determinations

MIC evaluations were performed in Mueller–Hinton broth in accordance with CLSI guidelines, following overnight incubation at 37 °C. All tests were performed in triplicates [13].

### 2.5. H-Uracil Proliferation Assay

Extracellular bacteria (2 × 10^6^ cells/mL) were distributed into 96-well flat bottom plates (Nunc; ThermoFisher, Bremen, Germany) in triplicates and incubated for 3 days at 37 °C/5% CO_2_. Next, ^3^H-Uracil (0.3 µCi/mL, Biotrend, Köln, Germany) was added overnight at 37 °C/5% CO_2_. Mtb were then fixed and killed with 4% paraformaldehyde (PFA) at room temperature (RT) for 20 min, and were then harvested (Cell harvester; Inotech, Derwood, MD, USA) onto a filtermat (Perkin Elmer, Waltham, MA, USA). Afterwards, wax plates (Meltilex A; Perkin Elmer) containing scintillation liquid were molten onto the mats. Samples were measured with a beta counter (Hidex sense micro beta counter; Turku, Finland) and the mean values of the triplicates were calculated.

### 2.6. Effect of Peptides on Zika Virus Infection

Virus stocks of ZIKV MR766, a ZIKV strain isolated from a sentinel rhesus macaque in 1947 [14] were generated by inoculating 70% confluent Vero E6 cells in 175 cm^2^ cell culture flasks for 2 h, before adding a 40 mL medium. After 3–5 days, the virus was collected by centrifuging the cell supernatant to remove cell debris for 3 min at 330× *g*. Virus stocks were stored at −80 °C. To determine the ZIKV infection, 6,000 Vero E6 cells were seeded into 96-well plates the day before. Peptides were dissolved in 3% DMSO and centrifuged for 10 min at 14,000× *g*. ZIKV was mixed 1:1 with 0, 33.3, 100, and 300 µg/mL of the peptides or PSVBS, and 3% DMSO [15] as controls, and incubated for 2 h at 37 °C, before adding 20 µL of the mixture on cells. Two days later, infection rates were determined with a cell-based ZIKV immunodetection assay [16,17]. Cells were washed with Phosphate Buffer Saline (PBS) and fixed with 4% paraformaldehyde (PFA) for 20 min at room temperature. Cell permeabilization was performed with cold methanol for 5 min at 4 °C and the cells were then washed with PBS. Afterwards, the cells were incubated with mouse anti-flavivirus antibodies 4G2 in antibody buffer, for 1 h at 37 °C, washed 3 times with washing buffer, and incubated with an HRP-coupled anti-mouse antibody (1:20,000) for 1 h at 37 °C. After 4 washing steps with PBS, the TMB substrate was added. After an incubation of 5 min at room temperature, the reaction was stopped with 0.5 M sulfuric acid, and absorption was measured at 450 nm, and the baseline was corrected at 650 nm, using an ELISA microplate reader (Molecular Devices, Silicon Valley, CA, USA). Values were corrected for the background signal derived from the uninfected cells and the untreated controls were set to 100% infection.

### 2.7. Effect of Peptides on HSV Infection

The Herpes-Simplex-Virus 2 (Strain 333) was kindly provided by Patricia Spear (Northwestern University, Evanston, IL, USA). The virus was propagated as described for ZIKV. Supernatants were centrifuged for 3 min at 330× *g*, to remove cellular debris, and then aliquoted and stored at −80 °C as virus stocks. To determine the HSV-2 infection, 5000 ELVIS cells were seeded the day before into 96-well-flat-bottom plates. Before infection, the cell medium was removed and 80 μL of X-vivo cell medium supplemented with 2 mM L-glutamine, 100 units/mL penicillin, and 100 μg/mL streptomycin was added. Peptides were dissolved in 3% DMSO and centrifuged for 10 min at 14,000× *g*. Experiments were done by mixing 35 μL of the peptide samples in concentrations of 0, 33.3, 100, and 300 µg/mL of the peptides or PSVBS, and 3% DMSO as controls, with 35 μL of HSV-2 for 2 h at 37 °C. Then, 20 μL of the peptide-virus mix were added to each well. One day post-infection, the medium was discarded and 40 μL of diluted Gal-Screen^®^ substrate/buffer A (1:4 in PBS) (ThermoFisher Scientific, Waltham, MA, USA) was added for 30 min of room temperature incubation. Then, 35 μL were transferred into white 96-well plates and the substrate conversion was measured as relative light units per second, using the Orion II Microplate Luminometer. Values were corrected for the background signal derived from the uninfected cells.

### 2.8. Effect of Peptides on SARS-CoV-2 Pseudo Particles

#### 2.8.1. Pseudotyping of VSV with SARS-CoV-2S

Viral pseudoparticles based on the vesicular stomatitis virus containing the Spike protein of SARS-CoV-2 were produced, as previously described, with minor modifications [18]. HEK293T cells were first transfected with pCG1-SARS-2-S (kindly provided by Stefan Poehlmann, German Primate Center, Göttingen, Germany), using Transit LT-1 (Mirus, #MIR2306) and one day later, transduced by replication-deficient VSV*ΔG_fluc_eGFP pseudotyped with VSV-G (kindly provided by Gert Zimmer, Institute of Virology and Immunology, Mittelhäusern, Switzerland) [19]. After 1 h of incubation, the inoculum was removed, cells were washed 3× with PBS, and a fresh medium was added. After 16 h, supernatants were harvested and centrifuged to pellet cellular debris. The supernatant from hybridoma cells expressing anti-VSV-G antibodies (I1, mouse hybridoma supernatant from CRL-2700; ATCC) was added to the clarified pseudoparticle stocks at 1:10 (*v*/*v*) ratio, to block residual VSV-G-containing particles, before concentrating the supernatants 10-fold by volume, using the 100 kDa MWCO Amicon Ultrafiltration columns (Sigma-Aldrich, # UFC910096, St. Louis, MO, USA), aliquoting and storing at −80 °C until use.

#### 2.8.2. Transduction Experiments with SARS-CoV-2 Spike-Pseudoparticles

Transduction experiments with pseudoparticles were performed on Caco-2 cells. In brief, 10,000 Caco-2 cells were seeded onto the 96-well-flat-bottom plates, one day prior. Peptides were incubated with pseudoparticles at the indicated concentrations for 1 h at 37 °C, before adding the mixtures to cells at 1:10 dilution. Sixteen hours post-transduction, the medium was removed and the cells were washed once with PBS, before adding the Luciferase Cell Culture Lysis Reagent (Promega #E1531, Madison, WI, USA). The lysates were then transferred to white 96-well plates, 50 μL Luciferase Assay System Reagent (Promega #E1500, Madison, WI, USA) was added and substrate conversion was measured as relative light units per second, using the Orion II Microplate Luminometer. The values were corrected for the background signal derived from the untransduced cells.

#### 2.8.3. Cell Viability Assay

Vero and ELVIS cells were seeded, as described above in the infection experiments. The next day, peptides were added at the indicated concentration and cell viability was quantified after 48 h, with the MTT (3-(4,5-dimethylthiazole-2-yl)-2,5-diphenyl tetrazolium bromide,)-based assay. In brief, the medium was removed and 90 μL PBS and 10 μL MTT (5 mg/mL in PBS, Sigma-Aldrich, St. Louis, MO, USA) solution were added per well. Following a 2.5 h incubation time at 37 °C, the supernatant was discarded and the formazan crystals were dissolved in 100 μL 1:1 DMSO-EtOH solution. Absorption was measured at 450 nm and the baseline was corrected at 650 nm, using a Vmax kinetic microplate reader (Molecular Devices, Silicon Valley, CA, USA). The untreated controls were set to 100% viability. For cytotoxicity evaluation on Caco-2 cells, CellTiter-Glo^®^ Luminescent Cell Viability Assay (Promega #G7571, Madison, WI, USA) was used as instructed by the manufacturer, with minor modifications. The medium was removed from cells, CellTiter-Glo reagent (diluted 1:1 with PBS) was added and incubated for 10 min at RT before transferring the lysates to white 96-well plates and measuring the luminescence as relative light units per second, using the Orion II Microplate Luminometer. The untreated controls were set to 100% viability.

## 3. Results

For more comprehensive antimicrobial examinations, the three Cm-p5 peptides were chemically resynthesized in large scale amounts (>100 mg batches) and were properly folded and analyzed, as described previously [3].

### 3.1. Antibacterial Activity

The antibacterial activity of the antimicrobial peptide Cm-p5, the cyclic- monomer and the two associated dimers (Figure 1) against *Streptococcus agalactiae*, *Staphylococcus aureus* MRSA, *Klebsiella pneumoniae* ESBL, *Acinetobacter baumanii*, and *Enterococcus faecium* (VRE) in the Agar overlay assay is shown in Table 1. As expected and demonstrated before [3] (results not shown), unmodified Cm-p5 was unable to exert antibacterial activity. On the other hand, the monomer and two dimers showed activity against *K. pneumoniae* ESBL, *Acinetobacter baumanii*, and *Enterococcus* faecium (VRE). While the peptides showed activity against *K. pneumoniae* ESBL only at 100 and 1000 µg/mL, *A. baumanii* and *E. faecium* were sensitive to all concentrations of the derivatives that we tested. However, only the cyclic monomer was able to inhibit *S. aureus* MRSA.

Next, we determined the antibacterial MIC of Cm-p5 derivatives against the Gram-positive strain *S. agalactiae* and the Gram-negative species *K. pneumoniae* ESBL and *P. aeruginosa* PA14. In this experiment, none of the Cm-p5 derivatives exhibited a significantly antibacterial activity against *K. pneumoniae* ESBL and *S. agalactiae*. However, dimer 2 was moderately active against *P. aeruginosa* PA14, with an MIC of 141.3 µg/mL.

### 3.2. Antimycobacterial Activity

**Figure 2:**
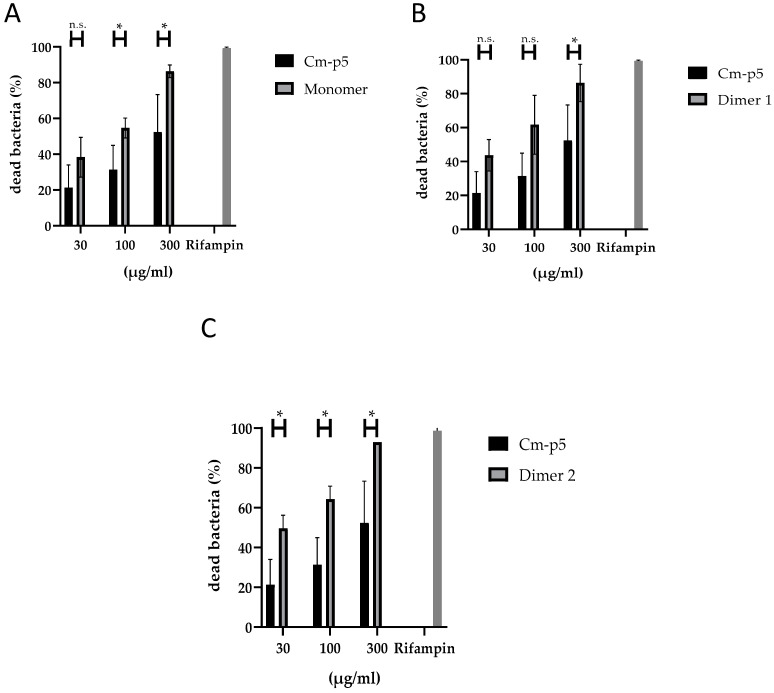
Antimycobacterial activity of Cm-p5 and its derivatives Monomer, cyclic (**A**), Dimer 1 (**B**) and Dimer 2 (**C**) at different concentrations (30 µg/mL, 100 µg/mL, 300 µg/mL) on virulent extracellular *Mycobacterium tuberculosis* was determined using ^3^H-uracil proliferation assay. Bacteria were incubated in the presence of AMPs for 3 days and an additional day with ^3^H-Uracil. Rifampin (2 µg/mL) was used as a positive control. Experiments were performed in triplicates and the results are shown as mean values of three independent experiments. Statistical significance was calculated using the Wilcoxon rank-sum test (n.s. = not significant, * = *p* ≤ 0.05).

The antimycobacterial activity of Cm-p5 derivatives on virulent extracellular Mtb is shown in Figure 2. All derivatives exhibited antimycobacterial activity in a concentration-dependent manner. Nevertheless, Dimer 2 (antiparallel) showed the highest activity among the derivatives with 50% antimycobacterial activity at 30 µg/mL.

### 3.3. Antiviral Activity

**Figure 3:**
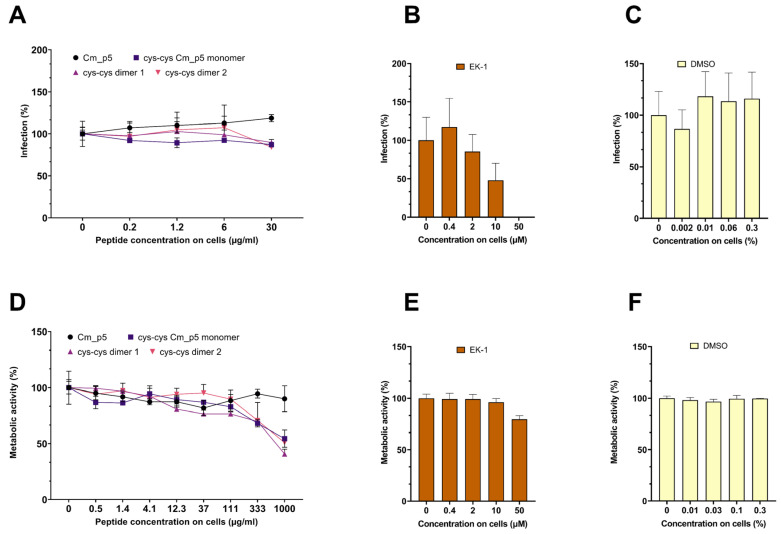
Effect of Cm-p5 peptides on SARS-CoV-2 pseudoparticle (PP) transduction. (**A**,**C**) Luciferase-encoding PP harboring the SARS-CoV-2 Spike protein were incubated with the peptides or the DMSO control for 1 h at 37 °C. Thereafter, the mixtures were inoculated on Caco2 cells, resulting in the indicated concentrations of the peptides on cells. The infection rates were determined 16 h post transduction by measuring the cell-associated luciferase activity. Shown above are the mean values derived from triplicate infections ± SD (relative to controls containing no peptide, 100%). (**B**) Caco2 cells containing the indicated concentrations of the SARS-CoV-2 inhibitory peptide EK1 were inoculated with SARS-CoV-2 PP, and the infection rates were determined as described under (**A**). (**D**–**F**) Metabolic activity of the Caco2 cells incubated with peptides for 48 h. The metabolic activity of the cells was determined by an MTT assay (**D**,**F**) or CellTiter-Glo® Luminescent Cell Viability Assay (**E**). The effect of the Cm-p5 compound was measured in an independent experiment, but are shown in the same figure. Values shown are the mean values derived from triplicate measurements ± SD (relative to controls containing no peptide, 100%).

First, we determined a possible antiviral effect of the peptides against viral pseudoparticles (PP) that carry the spike protein of the pandemic SARS-CoV-2. For this, luciferase-encoding SARS-CoV-2 Spike PP were treated with peptides at concentration up to 300 µg/mL, and then the Caco2 cells permissive for SARS-CoV-2 were inoculated. Infection rates were determined 18 h later, by quantifying the luciferase activities in cellular lysates. As shown in Figure 3A, none of the peptides suppressed SARS-CoV-2 Spike PP infection. In contrast, the SARS-CoV-2 entry inhibitor EK1, a peptide that prevents the formation of the 6-helix-bundle in the Spike protein [20,21], suppressed SARS-CoV-2 PP infection in a concentration-dependent manner (Figure 3B), whereas DMSO showed no effect on infection (Figure 3C). At concentrations >300 µg/mL, the three Cm-p5 derivatives showed some cytotoxicity to the Caco2 cells (Figure 3D), the reference inhibitor EK-1 showed some toxicity at the highest concentration tested (50 µM; Figure 3E) but DMSO did not show any toxicity in the concentrations used (Figure 3F).

**Figure 4:**
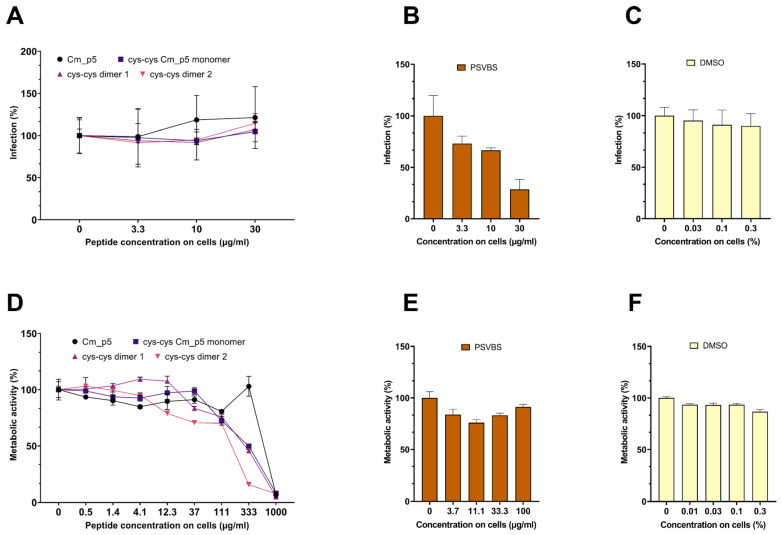
Effect of Cm-p5 and derivatives on ZIKV infection. (**A**–**C**) ZIKV was incubated for 1 h at 37 °C with the peptides, DMSO control, or the polyanionic ZIKV inhibitor PSVBS. Thereafter, Vero cells were inoculated with the mixtures (resulting in the indicated concentration of the peptides on cells) and the viral infection rates were determined 2 days later, by quantifying the viral E protein by in-cell ELISA. Shown are mean values derived from triplicate infections ± SD (relative to controls containing no peptide, 100%). (**D**–**F**) Metabolic activity of Vero cells that were incubated for 2 days with the indicated concentrations of peptides and controls. Cell viability was determined by the MTT assay. The effect of the Cm-p5 compound was measured in an independent experiment, but are shown in the same figure. The values shown are the means derived from triplicate measurements ± SD (relative to the controls containing no peptide, 100%).

Next, we evaluated the possible antiviral effects of the peptides against ZIKV (Figure 4). For this, the virus was exposed to the peptides or PSVBS, a polyanionic polymer that was previously shown to inhibit the ZIKV infection [15]. Infectivity of virus/peptide or virus/PSVBS mixtures was then assessed by inoculating the Vero cells and the infection rates were determined two days later, by quantifying the amount of intracellular viral E protein by in-cell ELISA [16,17]. As shown in Figure 4A, none of the peptides reduced virus infection at concentrations up to 30 µg/mL during virion treatment. In contrast, PSVBS inhibited ZIKV infection in a concentration-dependent manner (Figure 4B) and DMSO had no significant effect on infection rate in the concentrations used (Figure 4C). At high concentrations >300 µg/mL, the three Cm-p5 derivatives showed some cytotoxicity to the Vero cells (Figure 4D), whereas, the reference inhibitor PSVBS (Figure 4E) or DMSO in the concentrations used (Figure 4F) showed significant toxicity at the highest concentration tested.

**Figure 5:**
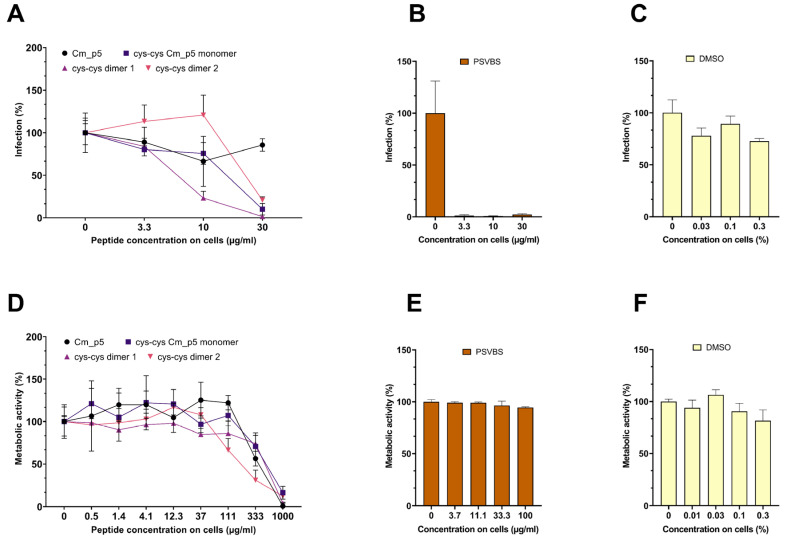
Cm-p5 derivatives inhibit the HSV-2 infection. (**A**–**C**) An HSV-2 stock was incubated with the peptides and controls for 30 min at 37 °C. Then, the ELVIS reporter cells were infected (resulting the indicated peptide concentrations on cells). The infection rates were determined one day later by measuring the cell-associated β-galactosidase activity in cellular lysates. Shown are the mean values derived from triplicate infections ± SD (relative to controls containing no peptide, 100%). (**D**–**F**) Metabolic activity of the ELVIS cells that were incubated for one day with the indicated concentrations of peptides and controls. Cell viability was determined by MTT assay. The values shown are means derived from triplicate measurements ± SD (relative to controls containing no peptide, 100%).

Finally, we analyzed whether Cm-p5 and its derivatives might affect the Herpes-Simplex Virus 2 (HSV-2) infection (Figure 5). For this, the virus was treated with peptides at concentrations up to 300 µg/mL for 2 h at 37 °C. Viral infectivity was determined by inoculating the ELVIS reporter cells that express β-galactosidase upon infection with HSV-1 or HSV-2 [12], the cells were incubated for 48 h with the peptides and the controls. As shown in Figure 5A, the parental Cm-p5 peptide did not affect the HSV-2 infection. Interestingly, the three derivatives resulted in a concentration-dependent inhibition of the HSV-2 infection with IC_50_ values of ~144 µg/mL for the cys–cys monomer, ~63 µg/mL for the dimer 1, and 275 µg/mL for dimer 2 (Figure 5A). Notably, at the highest concentration, dimer 1 almost completely inhibited the HSV-2 infection. PSVBS displayed potent antiviral activity and completely inhibited infection at 33 µg/mL (Figure 5B), whereas DMSO at the concentrations used showed no effect (Figure 5C). The Cm-p5 compounds did not reduce metabolic activity of ELVIS cells at concentrations < 100 µg/mL, but showed cytotoxicity at higher concentrations (Figure 5D). PSVBS and DMSO did not exert toxic effects at the concentrations tested (Figure 5E,F).

## 4. Discussion

In recent years, the emergence of a resistant microbial strain was observed against almost all classes of antimicrobials. In the current situation, novel antimicrobial agents are the only alternative to overcome this problem. One option are AMPs that were studied in great detail in the past decades and were approved for use [22]. However, the rational design of AMPs using natural occurring AMPs as a template is an alternative strategy with promising results. This approach overcomes various limitations of natural AMPs including reduced systemic toxicity and enhanced activity in blood/serum [23]. Considering these advantages, we evaluated the antimicrobial action of three derivatives of the antifungal peptide Cm-p5 against multiresistant and problematic bacterial strains and some further major human pathogens.

We previously evaluated these Cm-p5 derivatives against nonresistant bacterial and yeast strains; and they showed an improved antimicrobial action [3]. Therefore, we investigated the activity of these peptides against various multiresistant bacteria, including *S. aureus* MRSA, *K. pneumoniae* (ESBL), *Acinetobacter baumanii*, *Pseudomonas aeruginosa* and *Enterococcus faecium* VRE, all of which belong to the so-called ESKAPE pathogens, which are the cause of major problems in nosocomial infections worldwide [24]. *K. pneumoniae* (ESBL) is a multiresistant microorganism that is endemic in many communities and hospitals worldwide [25]. On the other hand, *S. aureus* MRSA is constitutively resistant to all β-lactam antibiotics and form part of the world health organization (WHO) “Global Priority List” of bacteria that represent the greatest threat to human health [26]. *P. aeruginosa* is another bacterium in this list, which causes severe infections with high morbidity and mortality in healthcare settings [27], but *P. aeruginosa* PA14 is a special strain with a high virulent potential [28]. Additionally to these multiresistant bacteria, we investigated the activity of the peptides against *S. agalactiae*, which is an important pathogen for pregnant women and children and causes severe septicemia and neonatal death. Moreover, it can cause diseases such as meningitis, septicemia, abscesses, infections in the urinary tract, and arthritis, particularly in immunocompromised adults [29,30].

The unmodified parent peptide Cm-p5 did not exhibit any antibacterial activity, which we expected in accordance with previous results [31]. Different to Cm-p5, its derivatives exhibited different degrees of antimicrobial activity against various bacterial species, including highly resistant strains like *E. faecium* VRE and *K. pneumonia* ESBL (Table 1). All derivatives showed antibacterial activity in agar diffusion assay at 1000 µg/mL against 6 of the 7 bacterial strains tested (Table 1). At concentrations of 20 µg/mL, the dimer 1 and dimer 2 still inhibited 4 or 5 of the 7 bacterial strains tested, respectively. In the same assay, LL-37 showed a potent activity against 5 of the 6 bacterial strains tested at a concentration of 1 µg/mL. The agar diffusion assay was influenced by a number of variables, including the diffusion of the antibiotic compound into the agar [32]. Some AMPs cannot spread very well in the agar, for this reason, we did an MIC determination using the broth dilution method (see Table 2). In this experiment, dimer 2 was the most active peptide, as demonstrated by an MIC value of 12.5 µg/mL against *L. monocytogenes* and a MIC value of 25 µg/mL against *A. baumanii* and *E. faecium VRE*. On the other hand, none of the Cm-p5 derivatives exhibited an antibacterial activity at 100 µg/mL against *K. pneumoniae* ESBL and *S. agalactiae*. Compared to the MIC values determined for LL-37 under similar conditions—depending on the bacterial strain—the resulting MIC values were within a range of 14–224 µg/mL [33]. Our results demonstrated that Cm-p5 derivatives were moderately active against multiresistant bacterial strains and mainly against the Gram-negative ones.

Tuberculosis (TB) is a reemergent disease due to the appearance and spread of multidrug-resistant strains (MDR-TB) [34]. Traditionally the treatment of TB was based on four drugs—isoniazid, rifampicin, pyrazinamide, and thambutol, but currently exists as a strong necessity for new anti-tuberculosis candidates. In last decades, many studies established that several AMP exert activity against Mtb [35]. Some peptides like Nisin S, a bio-engineered hinge derivative of Nisin A, have a potential effect over growth of Mtb, when compared to Nisin A [36]. It was reported that human β-defensins have anti-mycobacterial action and their analogues, such as hBD-Pep4, even showed an increase activity [37].

Design of the peptide derivatives is a useful strategy to generate potential antimicrobial agents. In this study, we were able to show that the synthesis of the monomeric analogue and the two dimers of Cm-p5 led to an increase in the activity against virulent extracellular Mtb, as compared to the parental peptide. The effect adds up to a two-fold increase in activity, even at low concentrations (30 µg/mL).

For many viral pathogens, no approved drugs exist (SARS-CoV-2, ZIKV) or current treatment options are not optimal because of side effects, low efficacy, or development of resistance (HSV-2). In this context, we evaluated the antiviral action of Cm-p5 derivatives against SARS-CoV-2, ZIKV, and HSV-2. All these viruses are enveloped, and could thus be targeted by peptides that act via interaction with the lipid membrane, regardless of the nucleic acid nature of the viral genome [38].

We did not observe antiviral effects of Cm-p5 and its derivatives against the ZIKV and SARS-CoV-2 pseudoparticles, at concentrations of up to 300 µg/mL. However, the three optimized derivatives showed a concentration-dependent anti-HSV-2 effect. These data show that the Cm-p5 derivatives do not exert a broad-antiviral activity but rather seem to inhibit a specific viral pathogen. Whether this was indeed the case, or whether the derivatives might also inhibit related viruses, such as HSV-1 or other herpesviruses, remains to be analyzed. It might also be possible that the Cm-p5 derivatives might not target the viral envelope bilayer but rather a HSV-2 glycoprotein that is essential for viral attachment or entry. The observed antiviral activity of the most potent derivative is in the median µg/mL range, and thus is clearly too high for a systemic administration. However, it needs to be considered that HSV-2 is a sexually transmitted viral pathogen. Microbicides that aim to protect women form acquiring HSV-2 are applied topically onto the vagina mucosa, and creams or gels that contain mg/mL concentrations of antivirally active peptides can be easily formulated. Our experiments confirmed that cyclization of Cm-p5 improved their antimicrobial activity, specially the antibacterial one. This strategy was used by other authors such as Gongora-Benítez and colleagues. This group reported that cyclization of peptides could enhance the pharmacological properties (potency, selectivity, stability, bioavailability, etc.) of their linear counterparts [39]. Although our peptides showed some promising antimicrobial and antiviral activity, compared to other AMPs, it is lower and a potential toxicity is clearly visible using concentrations >100 µg/mL (Figure 3D, Figure 4D and Figure 5D), which is a major concern and limitation. We believe that our present results could lead to future research toward improvement of Cm-p5 potency and reduced toxicity of its derivatives.

## 5. Conclusions

We synthesized three Cysteine-containing derivatives of the snail-derived peptide Cm-p5. The cyclic monomer, as well as a parallel and an antiparallel dimer of Cm-p5, showed an improved activity against the multiresistant strains of bacteria, including a virulent extracellular strain of *Mycobacterium tuberculosis,* and against HSV-2. Since the gap between effective concentrations and the start of toxic concentrations is quite narrow, further optimization of these sequences needs to be carried out.

## Figures and Tables

**Table 1:** Agar diffusion test of Gram-positive and Gram-negative bacterial species against the antimicrobial peptides CMP5 cys-cys monomer and dimer 1 and dimer 2.

	Concentration	CMP5 cys-cys Monomer			CMP5 cys-cys Dimer1			CMP5 cys-cys Dimer2			LL-37
Bacterial Species		1000 mg/L	100 mg/L	20 mg/L	1000 mg/L	100 mg/L	20 mg/L	1000 mg/L	100 mg/L	20 mg/L	1 mg/L
*Streptococcus agalactiae*		0.8	0.4	-	1	0.7	-	1.1	0.6	0.3	n.d.
*Staphylococcus aureus* MRSA		-	-	-	-	-	-	-	-	-	0.1
*Listeria monocytogenes*		0.9	0.5	-	1.2	0.7	0.3	1.2	0.8	0.3	1.1
*Klebsiella pneumoniae* ESBL		0.6	-	-	0.7	0.5	-	0.9	0.5	-	0.8
*Pseudomonas aeruginosa*		0.8	0.4	-	1	0.7	0.3	1	0.6	0.3	0.8
*Acinetobacter baumanii* ATCC 19606		0.7	0.4	-	1	0.6	0.4	0.9	0.6	0.4	0.7
*Enterococcus faecium* VRE DSM 17050		0.9	0.6	0.4	1.1	0.8	0.5	1.1	0.8	0.5	1.0

Susceptibility against different concentrations of the three AMPs was tested by agar diffusion assay. A total of 10 µL of a stock solution at a concentration as indicated above was spotted on to an agar containing the respective bacterial species. After overnight incubation at 37 °C, inhibition zones around the tested AMP were measured in centimeters. Determinations were done in triplicates. LL-37 was used as a positive control. n.d.: not determined.

**Table 2:** MIC determinations of CMP5 cys-cys monomer and dimer 1 and dimer 2. MIC determinations were performed in the Mueller–Hinton broth in accordance with CLSI guidelines, following overnight incubation at 37 °C. All tests were performed in triplicates.

	MIC Values	AMP Monomer (MIC)	AMP Dimer 1 (MIC)	AMP Dimer 2 (MIC)
Bacterial Species				
*Streptococcus agalactiae*		>100 mg/L	>100 mg/L	>100 mg/L
*Listeria monocytogenes*		100 mg/L	50 mg/L	12.5 mg/L
*Pseudomonas aeruginosa*		>100 mg/L	>100 mg/L	>100 mg/L
*Klebsiella pneumoniae* ESBL		>100 mg/L	>100 mg/L	>100 mg/L
*Acinetobacter baumanii*		100 mg/L	50 mg/L	25 mg/L
*Enterococcus faecium* VRE		50 mg/L	50 mg/L	25 mg/L

## Data Availability

Data is contained within the article.
